# Deep-Learning-Based Recovery of Missing Optical Marker Trajectories in 3D Motion Capture Systems

**DOI:** 10.3390/bioengineering11060560

**Published:** 2024-06-01

**Authors:** Oleksandr Yuhai, Ahnryul Choi, Yubin Cho, Hyunggun Kim, Joung Hwan Mun

**Affiliations:** 1 Department of Bio-Mechatronic Engineering, College of Biotechnology and Bioengineering, Sungkyunkwan University, Suwon 16419, Republic of Korea; oleksandr@g.skku.edu (O.Y.); yubinc@g.skku.edu (Y.C.); 2 Department of Biomedical Engineering, College of Medical Convergence, Catholic Kwandong University, Gangneung 25601, Republic of Korea; achoi@cku.ac.kr

**Keywords:** motion capture and analysis, long-term missing data, multi-camera data integration, artificial intelligence, adaptive Huber loss, data augmentation

## Abstract

Motion capture (MoCap) technology, essential for biomechanics and motion analysis, faces challenges from data loss due to occlusions and technical issues. Traditional recovery methods, based on inter-marker relationships or independent marker treatment, have limitations. This study introduces a novel U-net-inspired bi-directional long short-term memory (U-Bi-LSTM) autoencoder-based technique for recovering missing MoCap data across multi-camera setups. Leveraging multi-camera and triangulated 3D data, this method employs a sophisticated U-shaped deep learning structure with an adaptive Huber regression layer, enhancing outlier robustness and minimizing reconstruction errors, proving particularly beneficial for long-term data loss scenarios. Our approach surpasses traditional piecewise cubic spline and state-of-the-art sparse low rank methods, demonstrating statistically significant improvements in reconstruction error across various gap lengths and numbers. This research not only advances the technical capabilities of MoCap systems but also enriches the analytical tools available for biomechanical research, offering new possibilities for enhancing athletic performance, optimizing rehabilitation protocols, and developing personalized treatment plans based on precise biomechanical data.

## 1. Introduction

Motion capture (MoCap) technology, which is a linchpin in the realm of dynamic movement analysis, has become indispensable across a wide range of applications, from artistically focused applications, where it is used to create enchanting visuals in cinema and animation, to precision-dependent fields, where it is used in biomechanics and surgical navigation [1,2,3]. In particular, optical-marker-based MoCap systems offer unparalleled spatial and temporal accuracy, as they capture the intricacies of human motion by triangulating the three-dimensional (3D) positions of markers from multiple two-dimensional (2D) perspectives [4]. The advent of optical-marker-based MoCap has galvanized the creative and scientific communities in a wide-ranging manner, with example applications including its use in enhancing the creation of lifelike animations, the analysis of athletic performance, the study of intricate biomechanical processes, and advancements in surgical techniques through enhanced navigational aids [3,4,5]. In entertainment, MoCap serves as the backbone for creating compelling and immersive experiences; in clinical settings, it aids in the diagnosis and rehabilitation of gait abnormalities; and in the athletic arena, it provides critical insights into performance optimization and injury prevention [6,7,8].

Despite these profound impacts, MoCap technology is not without its challenges. One such challenge is the phenomenon of data loss, which is a significant obstacle to the seamless application of MoCap. Such data loss typically arises from occlusions, i.e., cases in which a marker becomes obscured from the view of the cameras due to the natural movement of limbs, interactions with props, or environmental constraints [9]. Other factors that contribute to data loss include marker detachment, technical malfunctions, and limitations in the camera’s field of vision [5,9]. Such disruptions result in incomplete datasets, which compromise the integrity of the motion capture process and can lead to inaccurate analyses and interpretations [9,10]. In this context, various data recovery methods for MoCap have been proposed, each of which is tailored to overcome the challenges of data loss. These methods can be broadly categorized into two distinct groups based on their dependency on inter-marker relationships and predefined motion models.

Group 1 encompasses methods that utilize specific inter-marker relations and motion models. Although data-driven methods like local coordinate system interpolation, principal component analysis (PCA), and low rank matrix completion [11,12,13,14,15] are efficient, they may overfit, and they fail to capture the complexity of dynamic movements. Model-based methods, such as Kalman filter or the inverse-kinematics-based gap filling method [16,17,18], ensure physical consistency, but they are limited by rigid assumptions and struggle when faced with unexpected occlusions. While machine-learning-based methods show promise, particularly when using neural networks [19,20,21,22], they are hampered by their reliance on extensive training data and focus on skeletal data, indicating the inherited limitations tied to human body constraints. Recently, deep-learning-based methods, specifically leveraging LSTM architectures [20,21], have attracted attention for use in the recovery of missing data. LSTMs are favored for their proficiency in learning temporal dependencies, which is crucial for accurately predicting the sequence of movements in MoCap data. Despite their advantages, such models often require the use of substantial training datasets and may not effectively capture complex spatial relationships or sufficiently generalize across different movement types. Hybrid methods like linear dynamical systems (LDS) with constraints, low-dimensional Kalman smoothing, group-sparse low-rank (GS-LR), and probabilistic model averaging (PMA) [23,24,25,26,27,28] attempt to integrate the advantages of other approaches, but these all involve high computational complexity and require intensive parameter tuning.

These methods are fundamentally dependent on precise inter-marker relationships, meaning they require at least two markers with accurately known connections between them to be used, as well as specific predefined motion models [11,17,24,28]. While this dependency enhances data recovery accuracy in certain specialized scenarios, it simultaneously imposes significant limitations on the broader applicability of such methods. Specifically, these methods demand considerable effort, time, and computational resources [11,22] to define and maintain these exact inter-marker relationships and pre-defined motion models. Consequently, a model that has been calibrated for a particular skeletal structure or movement pattern may not yield accurate results when it is applied to different marker configurations or different types of motion [28]. This limitation becomes particularly pronounced in diverse applications where it is essential to use adaptable models, as well as when opportunities for training data for specific scenarios are either not readily available or are too constrained.

Meanwhile, Group 2 includes methods that treat the recovery of each marker’s data independently. This group includes techniques like cubic and Hermite interpolation [29], which provide quick fixes for short-term data gaps. However, they are also constrained by the quality of input data, and they may struggle with longer gaps or more complex motions. The sparse low-rank method [28] is adept at reconstructing data from highly incomplete datasets, but its sensitivity to the specific context of data loss may cause it to struggle with extended data loss periods [14,22].

Although the methods in Group 2 address the primary limitation of Group 1’s dependency on specific relationships and models, they also face their own set of challenges. For example, cubic spline interpolations, despite being independent of inter-marker constraints, may not effectively restore long-term missing MoCap data gaps [17] due to their inability to handle non-linearities and complex motion patterns that fall outside of their training data. As another example, although sparse low-rank methods are powerful for incomplete datasets, they may also falter when faced with extended data loss, thus necessitating a context-sensitive approach to reconstruction. Long-term missing MoCap data gaps represent a critical problem due to the associated loss of information, which can lead to significant inaccuracies in movement analysis [22]. This leads to difficulties arising from the need to infer data over extended periods, where traditional methods typically fail to capture the inherent dynamics of motion or the unique characteristics of individual markers.

With this background, the main purpose of the current research is to address the pervasive challenge of long-term data loss in multi-camera motion capture systems by using a novel recovery algorithm. The proposed U-net-inspired bi-directional long short-term memory (U-Bi-LSTM) autoencoder-based method is engineered to operate independently of marker relationships, thus making it versatile for various applications, including biomechanical analysis and surgical navigation. The methodological contributions of this paper are threefold: First, a preliminary stage is initiated in which multi-camera tracking data is newly augmented, missing data gaps are created, and initial imputation is conducted to refine the dataset. Next, the U-Bi-LSTM framework intricately processes the temporal and spatial complexities of MoCap data, and the final validation phase rigorously assesses the model’s accuracy and robustness across various motion capture scenarios. The main contributions of this paper are elaborated upon in the following:

1. Advanced data recovery deep learning model. At the core of this research is the newly proposed U-Bi-LSTM model, which is a sophisticated deep learning structure that is inspired by U-net and enhanced with bidirectional LSTM networks. This model is notable for its ability to intricately capture the temporal dependencies and spatial nuances that are inherent in MoCap data, thus representing a significant leap from traditional linear prediction models. A notable feature of this model is its integration of an adaptive Huber regression layer, which replaces classical regression approaches. This layer is specifically chosen for its robustness against outliers and effectiveness in minimizing reconstruction errors, particularly in scenarios involving long-term data loss. Complementing this advanced architecture, the proposed method begins with a preliminary phase utilizing piecewise cubic spline (PCS) interpolation or sparse low rank (SLR) matrix completion. This preliminary phase is critical in refining the integrity of the dataset, thereby setting a solid foundation for the deep learning process and ultimately ensuring enhanced accuracy in the reconstructed data.
2. Holistic multi-camera data synthesis and augmentation. The proposed algorithm uniquely integrates available multi-camera UV data as well as triangulated 3D (X, Y, Z) data, ultimately allowing for an all-encompassing reconstruction that meticulously considers inter-camera relationships. This holistic approach not only guarantees the fidelity of the recovered data but also establishes the U-Bi-LSTM as an all-encompassing solution for MoCap data recovery challenges. The algorithm also features a novel multi-camera approach to data augmentation: It meticulously augments UV tracking data from multi-camera systems, thus preserving the spatio-temporal relationships while adapting the augmentation process to align with specific camera resolutions. This method applies transformations such as translation, rotation, scaling, and noise addition in a systematic manner that maintains data uniformity and adherence to camera resolution constraints.
3. Rigorous validation. Lastly, the effectiveness of the proposed deep-learning-based method is thoroughly validated against datasets characterized by extensive missing data. In these validation scenarios, the model consistently demonstrates superior accuracy compared to reconstructions prepared using only PCS or the state-of-the-art SLR missing data recovery method. This superior performance underscores the model’s capability to significantly enhance traditional data imputation techniques. The algorithm was rigorously tested in various dynamic scenarios, including cases involving the recovery of lost data with diverse lengths and frequencies. These scenarios encompassed movements such as manipulating a calibration wand with varying speeds and patterns, performing arm abduction and adduction, hip flexion of different intensities, and movements occurring during surface registration using a surgical navigation probe. The robustness and versatility demonstrated in these tests underscore the algorithm’s applicability and potential impact across a wide range of MoCap applications.

## 2. Materials and Methods

**Figure 1:**
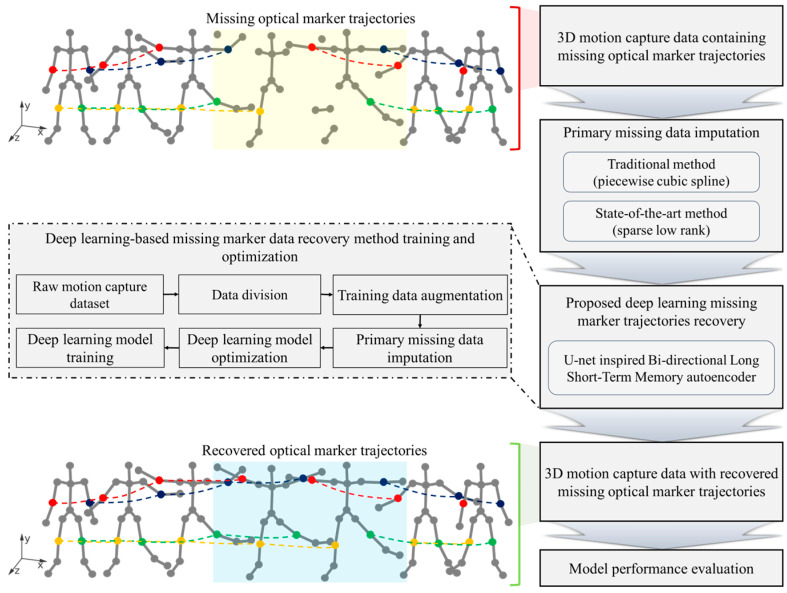
Comprehensive workflow for proposed deep-learning-enhanced multi-camera missing tracking data recovery method. The figure illustrates the process of capturing human movements and the occurrence of missing optical marker trajectories, demonstrated in the area highlighted in light yellow. Using the proposed deep learning method, these missing trajectories are accurately reconstructed, as shown in the area highlighted in light blue. The red and blue markers represent the RELB and LELB markers, respectively, while the yellow and green markers represent the RKNE and LKNE markers.

This section details the multi-faceted approach used in the present study to recover motion capture data that has been lost in multi-camera systems, including the data collection with precise calibration, experimentation capturing diverse movements, extensive data augmentation for robust training datasets, and simulation of realistic missing data for effective algorithm training and testing. This methodology integrates the traditional imputation methods—PCS and SLR—with an advanced U-Bi-LSTM network, which is an autoencoder that is designed for intricate data pattern recognition. The efficacy of the recovery process is scrutinized using reconstruction error by utilizing root mean square error (RMSE) metrics and wand length error. Figure 1 visualizes the detailed workflow, which encapsulates the entire process from the initial data capture to the final performance assessment, thus showcasing a cohesive strategy for data recovery.

### 2.1. Experiments

**Figure 2:**
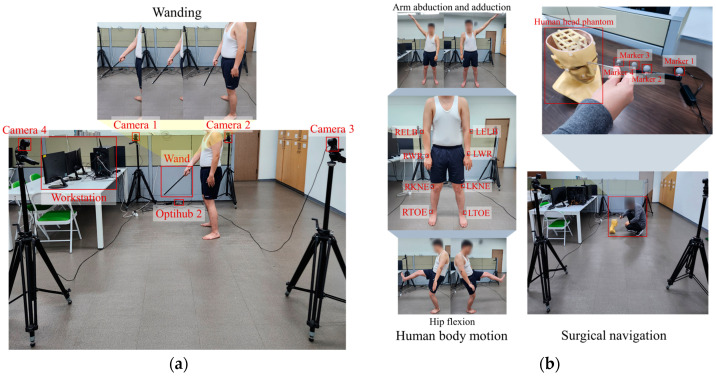
Experimental setup: (**a**) Schematics of the experimental multi-camera setup for MoCap data collection. (**b**) Marker placement for human body motion and surgical navigation validation experiments.

A strategic data collection setup was established to effectively capture and recover lost multi-camera MoCap tracking data. This setup included four synchronized CMOS cameras (OptiTrack FLEX: V100; Natural Point, Corvallis, OR, USA), each configured with a resolution of 640 × 480 and a frame rate of 100 Hz. These cameras were strategically positioned at intervals of 5 m to create an extensive field of view, as depicted in Figure 2a, to ensure maximal coverage for tracking. The data collection commenced with a meticulous multi-camera calibration process involving a three-axis frame and a calibration wand with known marker 3D coordinates and distances, along with the direct linear transformation (DLT)-based algorithm proposed by Shin et al. [4]. This setup guaranteed the precision required for the proposed method. After calibration, two male volunteers were recruited in generating ten distinct datasets. The first volunteer, aged 25 years old, had a height of 166 cm and a weight of 98 kg. The second volunteer, aged 26 years old, had a height of 173 cm and a weight of 68 kg. Before the experiment, subjects were informed about how to properly perform activities like wanding and surgical navigation, as well as exercises like arm abduction, adduction, and hip flexion, with a demonstration provided. During the exercises, each subject alternated between the right and left limbs for abduction, adduction, and hip flexion. The subjects gradually increased the exercise speed every 10 s, from the slowest to the fastest possible speed, according to their perceptions [30]. Each volunteer performed random movements with the calibration wand that were recorded for 50 s (equating to 5000 frames) within the carefully defined zone corresponding to the collective field of view of all cameras, as illustrated in Figure 2a. The accuracy of this data collection method was validated by comparing the triangulated distances between the markers on the calibration wand against their known distances, and this process yielded an average error margin of about 0.5 mm. This precision demonstrates the reliability of the multi-camera setup and the data collection process. Four of the five datasets that were randomly chosen from each of the two subjects were used as the training data for the deep-learning algorithm, while the remaining datasets were reserved for testing.

To further validate the proposed method’s performance and robustness, additional experiments involving human body motion tracking were conducted, such as arm abduction and adduction, hip flexion, and surgical navigation by performing surface registration.

1. Human body motion tracking. In total, four markers were attached to the bodies of two subjects, as shown in Figure 2b. Each subject performed arm abduction and adduction, and hip flexion exercises for 50 s, twice at varying intensities. During arm abduction and adduction, the markers on the right and left elbows and wrists (RELB, RWR, LELB, LWR) were tracked and used for validation. Meanwhile, during hip flexion, the markers on the knees and toes (RKNE, RTOE, LKNE, LTOE) were tracked and used for validation.

2. Surgical navigation experiment. Using a human head phantom [3] and a passive probe (NDI, Waterloo, ON, Canada), as depicted in Figure 2b, each subject carried out surface registration by tracing the probe over the phantom’s face for 50 s, twice at different intensities. Markers 1, 2, 3, and 4 on the probe, as demonstrated in Figure 2b, were used for validation in these surgical navigation exercises.

All experimental procedures were conducted while following the approved ethical guidelines set by the Ethics Committee of Sungkyunkwan University.

### 2.2. Data Augmentation

**Figure 3:**
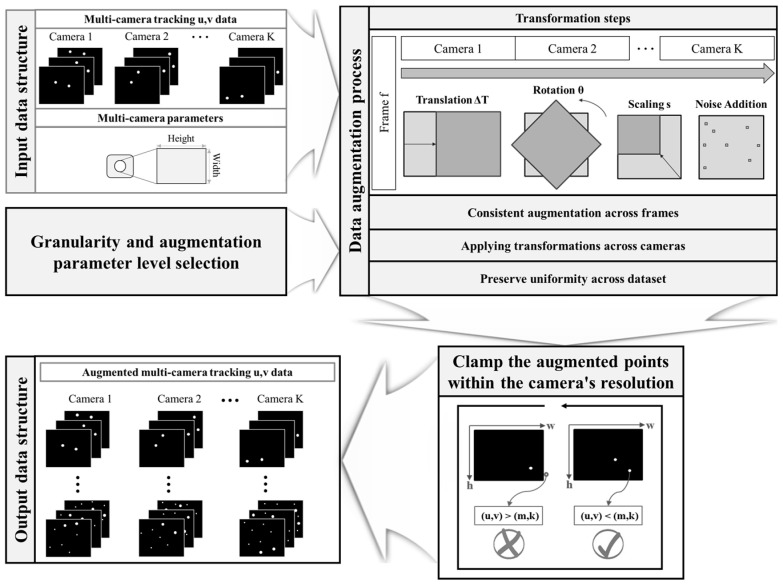
Schematic of the proposed data augmentation process for multi-camera motion tracking. First, collection of synchronized and preprocessed UV data from each camera occurs. Using the collected UV data and camera system information, such as resolution, determination of the augmentation parameters and their granularity is performed. The data is then augmented by applying transformations (translation, rotation, scaling, and noise addition) consistently across all frames and cameras. The augmented points are clamped within the camera’s resolution, considering the maximum width (m) and height (k). Any marker UV data that exceeds the camera’s field of view limits is eliminated. Finally, the remaining augmented UV data that stays within the entire camera’s field of view is used to generate augmented datasets ready for training the deep learning model.

The data augmentation process is a pivotal phase in preparing our training dataset, which is instrumental for the effective application of the proposed deep learning algorithm. Augmentation was meticulously applied to the multi-camera UV tracking data of the calibration wand in the manner described in the Experiments section herein. This process was designed not only to enhance the dataset with realistic variations but also to ensure that the necessary spatio-temporal fidelity for multi-camera MoCap systems would be achieved. As shown in Figure 3, the augmentation method was executed following an automated and systematic approach. Starting with synchronized and preprocessed UV data from each camera, the algorithm introduced transformations—translation, rotation, scaling, and noise addition—in a consistent manner across all frames and cameras. The transformation parameters were dynamically generated for each dataset iteration, involving small random translations (±0.05 units), rotations (±5 degrees), scaling factors (1 ± 0.02), and noise levels (±0.01 units) to simulate realistic and subtle variations in the camera capture conditions. These transformations were mathematically formulated to both respect the camera’s resolution boundaries and preserve the original data’s spatial and temporal relationships.

It was critical to adhere to the resolution limits to ensure that the augmented points did not fall outside of the camera’s field of view. As an example, a point $P_{original}$ translated by a vector ∆T=(∆u, ∆v) would be given by

(1) $$P_{new}=P_{original}+∆T$$

This would then be rotated around the origin by an angle *θ*, using the rotation matrix *R*, which is defined as

(2) $$R=\left[\begin{array}{cc} \cos(\theta ) & -\sin(\theta )\\ \sin(\theta ) & \cos(\theta ) \end{array}\right]$$

The rotated point $P_{rotated}$ is then scaled by a factor s. The scaling transformation, effectively altering the magnification of data points, was applied as

(3) $$P_{scaled}=P_{rotated}\times s$$

where the s was randomly selected within the range of 0.98 to 1.02, representing a ±2% change from the original size. Then, random Gaussian noise $N=\left(N_{u},\;N_{v}\right)$ is added to introduce variability. Finally, the augmented point $P_{augmented}$ is clamped within the camera resolution $C=\left(C_{u},\;C_{v}\right)$ as follows:

(4) $$P_{augmented}=\min\left(\max(P_{final},\;(0,\;0)),\;C\right)$$

Granularity—a measure of the fineness with which the transformation parameter space is sampled—was one of the key considerations in automatic augmentation parameter selection. It was used to determine the diversity of the augmentation by specifying the number of discrete levels within the permissible range for each transformation. Carefully calibrating the granularity allowed us to generate a spectrum of variations that enriched the training datasets while preserving the integrity of the motion data. In this study, the granularity was set at 10, meaning each transformation parameter (translation, rotation, scale, and noise) was selected 10 times randomly within their defined ranges.

By applying this rigorous augmentation method to our initial training datasets, we were able to increase the number of datasets that we could use for training by a factor of 100. This significant enhancement in the quantity and variety of training data is expected to directly contribute to increasing the proposed deep learning algorithm’s ability to learn and generalize across a comprehensive range of data loss conditions, thereby enhancing its performance in the recovery of lost tracking data.

### 2.3. Missing Data Simulation

To establish a robust foundation for evaluating the proposed deep-learning-based data recovery method, it was imperative to simulate realistic scenarios involving a loss of tracking data within a multi-camera MoCap system. This simulation process is critical for developing and testing the effectiveness of data recovery algorithms under conditions that closely mimic those that lead to real-world data loss due to occlusions, camera malfunctions, or environmental interferences. The simulation algorithm commenced with the initialization of parameters to dictate the number and duration of gaps within the tracking data. This was done to introduce data loss across the datasets in a uniform manner to ensure comprehensive training and testing of the recovery algorithm. A systematic approach was used to simulate gaps or missing data points in the UV tracking data captured by multiple cameras. The algorithm was designed to ensure that the object remains detectable at all times, preserving the basic level of data integrity necessary for successful recovery. Specifically, in this study, the fixed base level refers to the minimum visibility threshold across the camera network, maintained within a tracking volume defined by an intersection of the visible zones of all cameras, approximately measuring 4 m (length) × 3 m (width) × 2 m (height). This arrangement guarantees the minimal visibility of the object by at least one camera at all times, pivotal for robust multi-camera tracking and data recovery. For each simulated gap, the algorithm randomly selected a subset of cameras and a starting frame within the sequence, while taking care not to exceed the data bounds. A crucial validation step was executed to ensure that the object remained visible to at least one camera during the gap period. If these conditions were satisfied, the algorithm introduced missing values to represent missing data in the UV coordinates for the selected frames. This simulation was then extended to the corresponding XYZ data to align the 3D spatial information with the 2D planar gaps. The integrity of the 3D spatial data was adjusted based on the simulated 2D planar data, thus ensuring consistency across dimensions. This adjustment was considered to be vital for maintaining a long-term missing data scenario for data recovery testing, as the loss in UV data must be accurately reflected in the XYZ data to simulate real-world conditions under which it is not possible for 3D data to be reliably triangulated. For the purposes of the current study, which seeks to develop a method that is adept at recovering tracking data with long-term gaps, five gaps were randomly introduced in each training dataset. These gaps varied in duration, ranging from 100 to 900 frames, to ensure a proportional representation of different gap lengths. The simulation covered both markers on the calibration wand used during data collection, thus reflecting the diversity of the data loss scenarios involved. Approximately equal numbers of training datasets contained five gaps of each duration, thus indicating that a balanced environment for the proposed deep learning algorithm’s training and validation has been successfully established. This meticulous simulation process has allowed us to generate a comprehensive set of datasets that replicate the complexities of close to real-world data loss in MoCap systems, thus providing a solid foundation for the development and evaluation of advanced data recovery methods.

### 2.4. Missing Data Recovery Model

**Figure 4:**
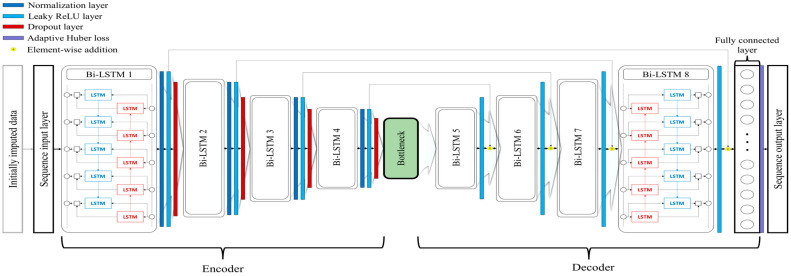
Schematic workflow of the proposed U-Bi-LSTM network.

Our approach for recovering missing tracking data in a multi-camera system follows a sequential methodology, starting with traditional data imputation methods before advancing to a sophisticated deep learning architecture. The entire process is depicted in Figure 4, which showcases the intricate U-Bi-LSTM network design.

#### 2.4.1. Primary Missing Data Imputation Methods

To begin, we employ general imputation methods to approximate the missing data points. This stage uses either the PCS method or SLR imputation according to the nature of the specific dataset.

Piecewise cubic spline missing data imputation method. The MATLAB (R2023a, MathWorks corp., USA) function *fillmissing* [31] is used here. This function applies spline interpolation column-wise, first across the *u* and *v* coordinates of each camera and then on the X, Y, and Z positions. The spline function can be mathematically expressed as

(5) $$S\left(x\right)=a_{n}{\left(x-x_{i}\right)}^{2}+b_{n}{\left(x-x_{i}\right)}^{2}+c_{n}{\left(x-x_{i}\right)}^{2}+d_{n}$$

where $S\left(x\right)$ represents the spline function, and where $a_{n}$, $b_{n}$, $c_{n}$, and $d_{n}$ are spline coefficients for the *i*-th interval. This method is particularly effective for non-linear data, as it ensures smooth continuity in the imputed data.

Sparse low-rank missing data imputation method. The SLR missing data imputation method proposed by Kamali et al. [28] employs an optimization framework to recover missing MoCap data. This technique separates multi-camera UV data and triangulated XYZ data, and it addresses them through a SLR matrix completion approach. The objective is to find matrices L and S that minimize the nuclear norm ${\left\|L\right\|}_{*}$ of L, which is the sum of its singular values to promote low-rank solutions, and the $L_{1}$ norm ${\left\|S\right\|}_{1}$ of S, the sum of the absolute values of its entries to encourage sparsity in the data matrix D. The regularization parameter λ, which is set within the range from 10^4^ to 10^5^, dictates the sparsity level of the solution, which affects both the accuracy and robustness of the reconstruction [28]. The SLR method is formulated as an optimization problem as follows:

(6) $$minimize\;{\left\|L\right\|}_{*}+\lambda {\left\|S\right\|}_{1},\;subject\;to\;D=L+S$$

Here, ${\left\|L\right\|}_{*}$ represents the nuclear norm of the matrix L. ${\left\|S\right\|}_{1}$ denotes the $L_{1}$ norm of the matrix S. Meanwhile, the parameter λ is a regularization parameter that controls the trade-off between these priorities of low-rank and sparsity.

#### 2.4.2. Deep-Learning-Based Missing Data Recovery Method

U-Bi-LSTM network. The proposed U-Bi-LSTM network, which is shown in Figure 4, is designed as an autoencoder, and it is specifically tailored for the recovery of missing data in multi-camera tracking systems. Initially, the dataset containing imputed missing data, which has been processed using traditional PCS or state-of-the-art SLR method, is transformed into an appropriate sequential format suitable for the U-Bi-LSTM network. For a single input dataset, this format is represented as (minimum sequence length) × (UV coordinates for each camera, and X, Y, Z coordinates). The core of the network consists of multiple bidirectional LSTM (Bi-LSTM) layers, each with varying numbers of hidden units (256, 128, 64, 32), ultimately leading to a bottleneck layer of size 16. This design enables the network to learn complex patterns in the data at different levels of abstraction [32]. The Bi-LSTM layers are formulated as follows:

(7) $${\vec{H}}_{t}=LSTM\left({\vec{X}}_{t},{\vec{H}}_{t-1};\;\vec{\Theta }\right)$$

(8) $${\overset{\leftarrow }{H}}_{t}=LSTM\left({\overset{\leftarrow }{X}}_{t},{\overset{\leftarrow }{H}}_{t+1};\;\overset{\leftarrow }{\Theta }\right)$$

(9) $$H_{t}=\left[{\vec{H}}_{t},\;{\overset{\leftarrow }{H}}_{t}\right]$$

Here, ${\vec{H}}_{t}$ and ${\overset{\leftarrow }{H}}_{t}$ are the hidden states at time t for the forward and backward passes, respectively; ${\vec{X}}_{t}$ and ${\overset{\leftarrow }{X}}_{t}$ are the inputs at time t for the forward and backward passes, respectively; and $\vec{\Theta }$ and $\overset{\leftarrow }{\Theta }$ are the parameters of the LSTM layers for the forward and backward directions, respectively. Each Bi-LSTM layer is followed by a layer normalization and a leaky ReLU activation function. The layer normalization ensures that the network’s internal features have a mean of zero and a standard deviation of one, thus enhancing the training stability [33]. The leaky ReLU function introduces non-linearity and allows for small gradients when the unit is not active, which enhances the network’s ability to learn complex patterns [34,35]. Dropout layers are interspersed throughout the architecture with a fixed rate of 0.5. These layers randomly deactivate some fraction of the neurons during training, thus preventing overfitting and enhancing the network’s generalization ability [36]. L2 regularization—consistently set to 0.001 across the network—is used to penalize large weights, with the aim of encouraging the development of simpler models that can generalize better to new data [37]. The architecture features a bottleneck layer of size 16 that acts as a compressed representation of the input data. The decoder part mirrors the encoder, with ascending numbers of hidden units (32, 64, 128, 256). It progressively reconstructs the data from the compressed representation provided by the bottleneck layer, with this reconstruction facilitated by the information that has been retained and carried over by the skip connections. The skip connections in the U-Bi-LSTM network are structurally akin to those found in U-net, which is known for its U-shaped architecture. This similarity is particularly evident in the way these connections bridge the gap between the encoder and decoder components of the autoencoder. The U-shaped architecture ensures a seamless flow of information, as it provides a pathway for context and details from the input layers to be directly funneled into the deeper layers of the network [38]. In the U-Bi-LSTM network, long skip connections are strategically placed to connect corresponding layers of the encoder and decoder. These connections bypass one or more intermediate layers to directly transmit information across the network. They are essential for preserving fine-grained details that might otherwise be lost in deeper layers. These skip connections are implemented through element-wise addition [39]. This method involves the direct summation of feature maps from an encoder layer to the corresponding feature maps in a decoder layer. The element-wise addition ensures that the essential features are retained and emphasized during the data reconstruction process. The final layer of the network is a fully connected the fully connected layer that integrates all the learned high-level features by performing a weighted sum across all nodes of the previous layer and then subsequently mapping this integration back to the original input dimensionality [37]. This process is essential for reconstructing the missing data by applying the learned transformations to the compressed feature representation from the bottleneck layer. The weights and biases of the fully connected layer are optimized through training to effectively translate the learned temporal patterns into a meaningful reconstruction of the original input sequence.

Adaptive Huber loss. The adaptive Huber loss function is a crucial aspect of the U-Bi-LSTM network due to its robust approach to handling prediction errors, particularly in the presence of outliers. This function adapts the traditional Huber loss [40], which is defined as a piecewise function transitioning from a quadratic to a linear form based on a predefined threshold in order to balance its sensitivity to errors of different magnitudes. For a set of predictions *Y* and targets *T*, the adaptive Huber loss *L* can be mathematically represented as

(10) $$L\left(Y,\;T\right)=\frac{1}{N}\sum_{j=1}^{n}L_{j}\left(Y_{j},\;T_{j}\right)$$

where $L_{j}$ indicates the individual loss for each prediction, computed by

(11) $$L_{j}\left(Y_{j},\;T_{j}\right)=\left\{\begin{array}{cc} 0.5\times {\left(Y_{j}-T_{j}\right)}^{2}, & if\;\left|Y_{j}-T_{j}\right|\;\leq \;\delta_{j}\\ \delta \times \left(\left|Y_{j}-T_{j}\right|-0.5\times \delta_{j}^{2}\right), & otherwise \end{array}\right.$$

where $\left|Y_{j}-T_{j}\right|$ is the absolute error for each element, $\delta_{j}$ is the adaptively determined transition point that dictates the shift from quadratic to linear behavior in the loss function, and N represents the count of predictions. Crucially, $\delta_{j}$, which is the adaptive transition point for each prediction, is dynamically calculated utilizing the median absolute deviation (MAD) from the prediction errors. MAD is a robust statistic that provides an estimate of variability, and it is less influenced by outliers compared to standard deviation [41]. It is defined as the median of the absolute deviations from the data set’s median:

(12) $$MAD=median\left(\left|X_{i}-median\left(X\right)\right|\right)$$

where $X_{i}$ are individual observations of the dataset X. *MAD* is particularly useful in datasets that may contain significant outliers, such as those obtained by multi-camera motion tracking systems. Consequently, the adaptive transition point $\delta_{j}$ can be determined as

(13) $$\delta_{j}=max\left(\alpha \times MAD\left(\left|T-Y\right|\right),\;\epsilon \right)$$

Here, α is a scaling parameter influencing the loss function’s sensitivity to outliers with the aim of ensuring it remains robust against extreme errors, and ϵ is a small constant guaranteeing the transition point does not reach zero, which maintains the stability and responsiveness of the loss function.

This adaptability of the Huber loss is particularly advantageous for datasets with notable outliers, as it offers a measured approach to penalizing errors. It allows the U-Bi-LSTM network to train in a stable manner and not be overly influenced by dataset deviations. This feature is pivotal in complex multi-camera tracking systems that are marked by various levels of noise and discrepancies. The integration of MAD into the adaptive Huber loss calculation ensures that the U-Bi-LSTM network accurately captures data trends and maintains high predictive accuracy, which is critical for achieving robust and generalizable performance in practical multi-camera tracking applications.

Ablation analysis. To investigate the individual and combined effects of various components of the U-Bi-LSTM network on the recovery of missing MoCap data, we conducted an ablation study [42] using wanding activity data. This study focused on comparing the following four network configurations: (1) Bi-LSTM with U-net components using adaptive Huber loss, (2) Bi-LSTM with U-net components using RMSE regression loss, (3) Bi-LSTM alone with adaptive Huber loss, and (4) Bi-LSTM alone with RMSE regression loss. This design allowed us to isolate the impact of each component and loss function on the overall performance of the network. Each variant was subjected to the same multi-camera missing data imputation process and trained under identical conditions to ensure a fair comparison. We then measured the model’s ability to reconstruct missing data points in the MoCap sequences. The data used in this study comprised sequences featuring artificially introduced gaps of various lengths and frequencies to simulate real-world data loss scenarios in multi-camera tracking systems. For each configuration, we recorded the missing data reconstruction error using root mean square error (RMSE), which quantitatively measures the average magnitude of the error between the predicted and actual values. A lower RMSE value indicates better performance in reconstructing the original data.

Hyperparameter optimization. In this study, we implemented a grid search methodology to systematically navigate through a predefined hyperparameter space to optimize the U-Bi-LSTM model’s performance. Grid search is an exhaustive method of searching through a manually specified subset of the hyperparameter space of a learning algorithm [43]. A grid search algorithm must be guided by some performance metric, such as missing data reconstruction RMSE, which is typically measured by cross-validation on the training set or evaluation on a held-out validation set. The hyperparameters we evaluated include input normalization techniques, the length of input sequences, the adaptive Huber alpha value, the maximum number of training epochs, mini-batch sizes, choice of optimizer, and the learning rate schedule. Mathematically, the grid search can be represented as

(14) $$Optimal\;hyperparameters={argmin}_{h\in H}\;CV\left(f\left(D_{train},\;h\right)\right)$$

where H is the hyperparameter space, CV denotes the cross-validation function, f represents the learning algorithm, $D_{train}$ is the training dataset, and h are the hyperparameters. For each hyperparameter set h, the performance metric CV was calculated. This metric is often calculated as the average of the validation scores obtained from the cross-validation process.

#### 2.4.3. Performance Measure

To objectively assess the performance of the proposed data recovery algorithms, i.e., PCS, SLR, PCS U-Bi-LSTM, and SLR U-Bi-LSTM, we implemented a rigorous validation framework. Tracking data were systematically occluded to simulate missing gaps across a variety of tool-based and human body-based MoCap activities. This simulation was applied to the datasets, each of which comprised 5000 frames collected from two volunteers and was intended to reflect a realistic spectrum of motion intensities and spatial trajectories. One of the main goals of this research is to develop a method that can be used to efficiently recover long-term missing data gaps, which typically have a length of more than 0.5 s, and which significantly increase the complexity of data recovery as they grow in length and number [44]. Specifically, missing data gaps were generated at intervals of 1 to 9 s (100 to 900 frames), representing different lengths of data absence. These intervals were introduced in each dataset with a frequency ranging from one to five non-overlapping gaps to evaluate the algorithms’ performance against both the length and the frequency of missing data. This strategy ensured that gaps would not be created at the dataset’s extremities to avoid the inherent limitations of the PCS method, which struggles with edge-located data loss. This procedure was iteratively conducted five times to calculate the missing data reconstruction error for each method, and an average was obtained from these calculations to minimize any variability introduced by differences in the nature of the tracking data in terms of gap location, outliers, or anomalies. The performance measure using the missing data reconstruction error for each method was determined based on the RMSE, which was calculated solely over the intervals of simulated data loss. This metric served as a standardized method for quantifying the accuracy of the data recovery process, and it can be expressed as follows:

(15) $$RMSE=\sqrt{\frac{1}{N}\sum_{i=1}^{N}{\left(O_{i}-R_{i}\right)}^{2}}$$

where $O_{i}$ represents the original data points, $R_{i}$ denotes the data points reconstructed using the evaluated method, and N is the number of data points within the missing data gaps.

For additional validation of the proposed method, wand length error, denoted as $L_{\mathrm{wand}\;\mathrm{error}}$, was calculated to measure the accuracy of the distance between two markers on the calibration wand. Upon reconstructing the 3D positions of the markers, the wand length error was computed as the absolute difference between the calculated Euclidean distance and the original known length, $L_{\mathrm{original}}$, between the markers. The wand length error over all n frames containing the recovered markers’ data can be expressed according to the following formula:

(16) $$L_{\mathrm{wand}\;\mathrm{error}}=\sum_{i=1}^{n}{\left|\sqrt{{\left(x_{2,i}-x_{1,i}\right)}^{2}+{\left(y_{2,i}-y_{1,i}\right)}^{2}+{\left(z_{2,i}-z_{1,i}\right)}^{2}}-L_{\mathrm{original}}\right|}_{2}$$

Here, $\left.|\cdot \right.{|}_{2}$ represents the Euclidean norm, and ($x_{1,i}$, $y_{1,i}$, $z_{1,i}$) and ($x_{2,i}$, $y_{2,i}$, $z_{2,i}$) denote the 3D coordinates of the first and second markers, respectively, for each *i*-th data point within the missing data gaps. This metric is particularly relevant because it directly reflects the physical accuracy of the motion capture system’s data recovery process, which is an approach that is often utilized to assess performance in related research due to its straightforward representation of spatial recovery accuracy [4]. The bone length between adjacent joints was not used as an additional performance measure due to the well-documented phenomenon of soft tissue artifact (STA), where markers affixed to the skin may not accurately reflect the motion of the underlying bone, thereby potentially introducing errors affecting the interpretation of skeletal movements [12]. This issue is particularly pronounced in high-mobility areas, where the relative movement of the skin to the bone can significantly affect the accuracy of joint angle calculations, and by extension, the inferred bone lengths. Consequently, focusing on wand length error allows for a more direct and unaffected measure of the recovery method’s accuracy while sidestepping the complexities introduced by STA.

To further substantiate the efficacy of the methods, we employed a paired *t*-test for comparative analysis. This statistical test was chosen to validate the significance of the performance improvements observed with the U-Bi-LSTM-enhanced methods compared to their traditional counterparts. A *p*-value of less than 0.05 was considered to be indicative of statistically significant differences, with such findings bolstering the assertion that the advanced algorithms provide a notable enhancement in data recovery, particularly in the context of long-term gap restoration. The entire methodology, including augmentation, data loss simulation, and model training and optimization, was executed using MATLAB while IBM SPSS Statistics (SPSS 29.0, IBM corp., USA) was used for performance evaluation and statistical analysis; all calculations were conducted on a computing machine equipped with an AMD Ryzen 7 3700X 8-Core CPU @ 3.60 GHz, an NVIDIA GeForce RTX 2080 Ti GPU 11 GB, and 48 GB RAM, running on the Windows 10 operating system.

## 3. Results and Discussion

**Figure 5:**
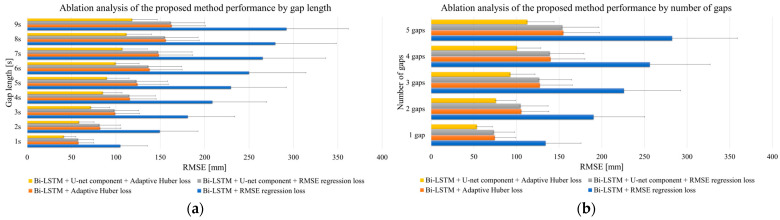
Ablation analysis of proposed method: (**a**) depending on the gap length; (**b**) depending on the number of gaps.

Results of ablation analysis. The ablation analysis, which was meticulously conducted over five iterations, unveiled significant insights into the performance enhancements afforded by each configuration. The detailed results are presented in Table S1 and visualized in Figure 5, which delineates the average results and the standard deviation (reconstruction error ± SD) for the restoration of the lost 3D coordinates of markers on the calibration wand for each of the four architectures evaluated in this study.

Notably, the results obtained from the ablation study, as extracted from the RMSE values, distinctly show that the architecture integrating Bi-LSTM with both U-net-inspired skip connections and an adaptive Huber loss function significantly outperformed the other examined architectures. It is imperative to mention that, in this rigorous examination, the input data for the proposed architecture did not undergo preliminary recovery with classical methods such as PCS or SLR. This was a deliberate methodological choice to ensure that the proposed model’s raw corrective capacity was authentically evaluated. The Bi-LSTM autoencoder was chosen as the foundational model for this investigation based on its adeptness at leveraging both preceding and subsequent data points within a sequence, which is pivotal for accurate prediction in sequential data analysis. Compared to the conventional unidirectional LSTM, the Bi-LSTM’s dual-directional learning framework enables the model to encapsulate a comprehensive temporal context, thereby enhancing its predictive accuracy for sequential data recovery tasks. References substantiating this preference include seminal works that highlight the superiority of Bi-LSTMs over single-direction LSTMs in various sequential data processing tasks [45,46]. Upon scrutinizing the outcomes presented in Figure 5, the effective impact of the U-net-inspired skip connections and the adaptive Huber loss function in the proposed Bi-LSTM autoencoder is evident. The U-net component introduces an innovative mechanism for feature retention across the depth of the network, which is particularly beneficial for complex patterns inherent in MoCap data. The adaptive Huber loss function—with its dynamic error sensitivity adjustment—enhances the robustness of the network, particularly when used with datasets characterized by anomalies or significant outliers. The results obtained herein necessitate a thorough, scientifically sound explanation of the contributions of the adaptive Huber loss and the U-net component to the model’s performance. The adaptive Huber loss amalgamates the advantages of L1 and L2 loss functions, thus introducing a conditional approach that is less sensitive to outliers and ultimately improving model reliability when faced with aberrant data. The U-net-inspired skip connections effectively facilitate the flow of gradient and information through the network, countering the vanishing gradient issue and aiding in the accurate reconstruction of the lost data. The deployment of these advanced architectural components results in a substantial increase in the model’s efficacy in reconstructing missing data.

Results of hyperparameter optimization. Upon completion of the grid search with fivefold cross-validation, we selected the optimal hyperparameters that minimized the reconstruction error of missing data in the MoCap sequences for the proposed U-Bi-LSTM-enhanced models. Table 1 presents the results of this optimization process while outlining the best-performing hyperparameters for each of the PCS U-Bi-LSTM and SLR U-Bi-LSTM models.

The results showed that z-score normalization was the most effective input normalization method for PCS U-Bi-LSTM, which is likely due to its ability to standardize feature scales and thus facilitate more stable and faster convergence during the training process. Similarly, rescale-zero-one normalization was considered to be optimal for SLR U-Bi-LSTM, possibly due to its effect on preserving the proportionate scaling of features. The chosen shorter input length (10) allowed for faster processing times while retaining sufficient temporal dynamics, balancing computational efficiency and model performance. An adaptive Huber alpha value of 0.01 was chosen for both models to maintain error sensitivity while reducing the influence of outliers. The mini-batch size was set to 32 for PCS U-Bi-LSTM and 64 for SLR U-Bi-LSTM, catering to the models’ computational requirements and balancing training speed. The optimization favored a moderate number of epochs (5), which suggests that the model was able to learn the essential patterns within a relatively small number of iterations without overfitting to the training data. The adam optimizer was preferred due to its adaptive learning rate capabilities, which is supported by the results of previous research, indicating its effectiveness in various deep learning scenarios [39]. The learning rate drop period of 10 and drop factor of 0.2 ensured that the learning rate decayed in a controlled manner, fine-tuning the model’s weights and biases. Overall, the results of the hyperparameter optimization underscore the importance of careful tuning when developing robust deep learning models, as the chosen parameters significantly impact model performance.

**Figure 6:**
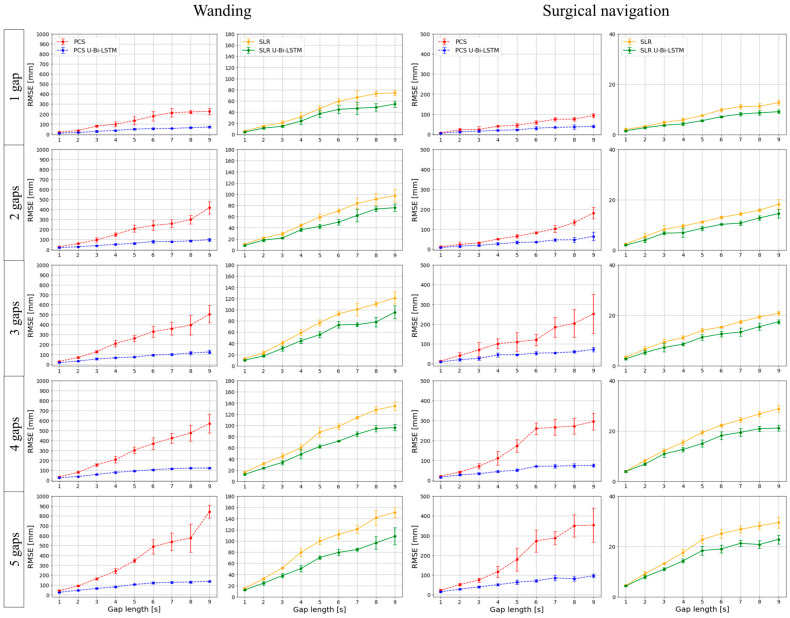
Multi-trial performance assessment of missing data recovery methods by gap length and frequency in tool-based MoCap activities.

**Figure 7:**
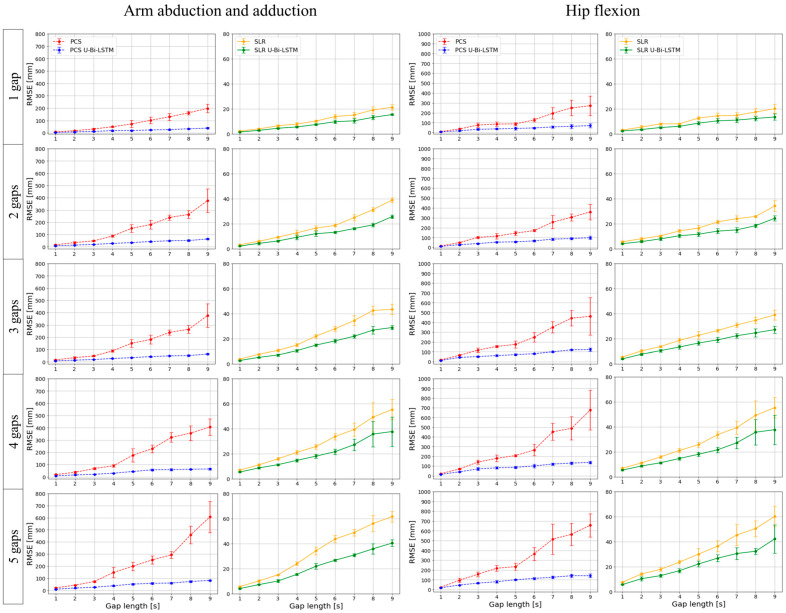
Multi-trial performance assessment of missing data recovery methods by gap length and frequency in human-body-based MoCap activities.

In this study, we empirically evaluated our MoCap data reconstruction methods under rigorous iterative testing. Each of the four methods—PCS, SLR, PCS U-Bi-LSTM, and SLR U-Bi-LSTM—was subjected to five iterations of validation to ensure the reliability and reproducibility of results. The missing data reconstruction RMSE for each method was calculated over each iteration, thus culminating in an aggregate analysis that was used in the plotting of Figure 6 and Figure 7.

For the tool-based MoCap activities (i.e., wanding and surgical navigation), our results—as depicted in Figure 6—demonstrate that both PCS U-Bi-LSTM and SLR U-Bi-LSTM outperformed their counterparts, regardless of the length or number of the missing data gaps. The proposed PCS U-Bi-LSTM showed a consistently lower missing data reconstruction RMSE compared to the conventional PCS, while the proposed SLR U-Bi-LSTM was superior to the state-of-the-art SLR method. Figure 7 extends the analysis described above to human-body-based MoCap activities (arm abduction and adduction, and hip flexion). The advanced U-Bi-LSTM models maintained a consistent trend of reduced RMSE values, thus indicating that our improvements were not confined to tool-based scenarios, as they also extended to complex human movements. The iterative approach and subsequent RMSE calculations provided a comprehensive view of the models’ capabilities, ultimately reinforcing the notion that the proposed U-Bi-LSTM-enhanced methods achieved strong performance under varying conditions and movement intensities.

**Figure 8:**
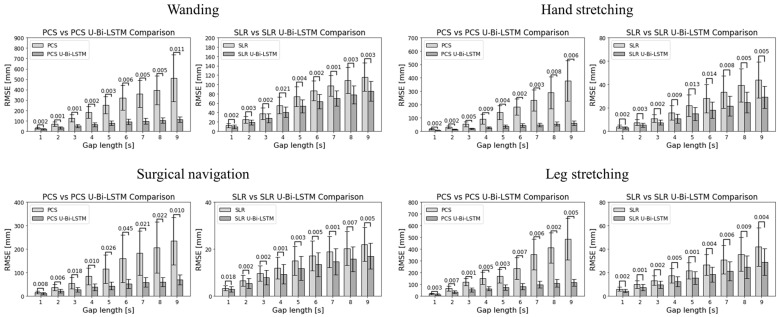
Paired *t*-test comparative analyses of MoCap data recovery methods performance by gap length.

**Figure 9:**
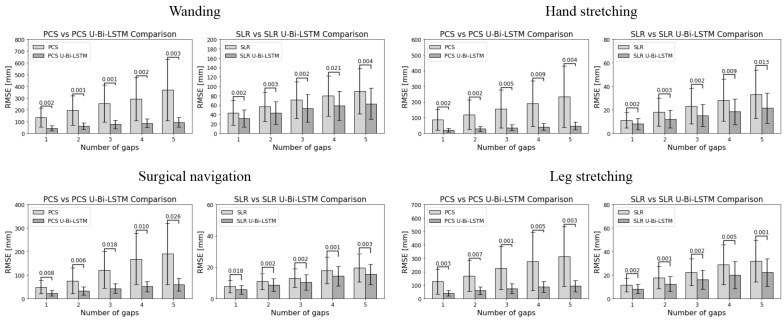
Paired *t*-test comparative analyses of MoCap data recovery methods performance by number of gaps.

Figure 8 and Figure 9 provide a visual representation of the performance of the different methods across all activities and gap metrics. The RMSE values were meticulously calculated for each gap length and gap number, where the PCS and SLR methods served as benchmarks against their advanced U-Bi-LSTM-enhanced counterparts.

By scrutinizing the results in Figure 8, it can be seen that in tool-based activities such as wanding, the PCS method exhibited a missing data reconstruction RMSE of 30.5584 ± 8.8504 mm for a 1 s gap, and that this was significantly reduced to 19.3654 ± 6.5213 mm with the application of the PCS U-Bi-LSTM method. Meanwhile, the SLR method, which showed an RMSE of 12.1592 ± 4.1677 mm for the same 1 s gap, demonstrated a decrease to 9.4656 ± 3.4081 mm with the application of the SLR U-Bi-LSTM method. As the gap length increased to 9 s, the RMSE for PCS rose sharply to 512.2272 ± 225.5896 mm, while the PCS U-Bi-LSTM method exhibited a more restrained increase, showing an RMSE of 110.6526 ± 26.7792 mm. The SLR method’s RMSE at a 9 s gap was 115.7212 ± 30.3819 mm, which was also outperformed by the SLR U-Bi-LSTM method’s RMSE of 86.0258 ± 21.1674 mm. Similar improvements were noted in the context of human-body-based activities. For example, during arm abduction and adduction exercises, the PCS method’s RMSE for a 1 s gap was 15.602 ± 4.5177 mm, compared to the PCS U-Bi-LSTM’s improved RMSE of 7.303 ± 1.9384 mm. Meanwhile, the SLR method’s RMSE was 3.9112 ± 1.3355 mm, which was further reduced to 2.848 ± 1.0633 mm with the SLR U-Bi-LSTM method. By similarly scrutinizing the results in Figure 9, it can be seen that with just one gap, in the same tool-based activities such as wanding, the PCS’s RMSE was 134.1418 ± 80.3905 mm, which was significantly higher than the PCS U-Bi-LSTM’s RMSE of 42.532 ± 21.3899 mm. Meanwhile, the SLR method’s RMSE at one gap was 43.6169 ± 26.2159 mm, and this was decreased to 31.6491 ± 18.598 mm with the SLR U-Bi-LSTM. As the number of gaps increased to five, the PCS method’s RMSE surged to 369.5683 ± 263.0945 mm, whereas the RMSE of the PCS U-Bi-LSTM method managed to remain at a more reasonable level of 92.8601 ± 40.3424 mm. Similarly, the SLR method’s RMSE for five gaps was 89.3563 ± 47.9494 mm, which was reduced in the SLR U-Bi-LSTM method to 62.7153 ± 33.2244 mm. Moreover, the bar graphs in Figure 8 and Figure 9 display the results of the paired *t*-test comparative analyses by gap length and gap number for all MoCap activities, where it can clearly be seen that both the PCS U-Bi-LSTM and SLR U-Bi-LSTM methods achieved significantly reduced RMSEs and standard deviations across all gap lengths and gap numbers. Altogether, the results revealed that PCS U-Bi-LSTM and SLR U-Bi-LSTM achieved statistically significant improvements (*p* < 0.05) over their general counterparts across all activities, gap lengths, and gap numbers.

**Figure 10:**
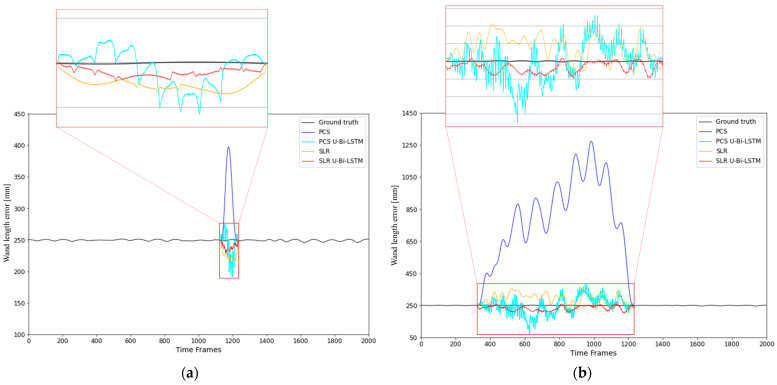
Comparison of the PCS, PCS U-Bi-LSTM, SLR, and SLR U-Bi-LSTM methods for the recovery of missing motion capture data in wand length tracking: analysis over (**a**) 100 frame and (**b**) 900 frame gaps.

An additional performance validation was conducted using the wand length error, and the results of this validation highlighted distinct differences in data recovery capabilities between the conventional and U-Bi-LSTM-enhanced methods, visually represented in Figure 10. In Figure 10, a direct comparison of the four data recovery methods—PCS, PCS U-Bi-LSTM, SLR, and SLR U-Bi-LSTM—is presented for tracking a missing data gap of 100 frames (Figure 10a) and 900 frames (Figure 10b) in wand length tracking.

In Figure 10a, which illustrates the recovery performance for a missing data gap of 100 frames, the U-Bi-LSTM-enhanced models (both PCS U-Bi-LSTM and SLR U-Bi-LSTM) delivered superior results compared to their conventional counterparts. While the PCS method showed a prominent spike far from the ground truth baseline and significant noise, PCS U-Bi-LSTM managed to reduce noise and maintains a closer trend. Similarly, SLR U-Bi-LSTM demonstrated a consistent recovery pattern with fewer fluctuations than SLR alone. Notably, the hybrid SLR U-Bi-LSTM model remained closest to the ground truth, indicating its effective management of long-range dependencies. In Figure 10b, the gap extended to 900 frames, which further tests the robustness of these models. PCS continued to struggle, with sustained deviations throughout the interval. PCS U-Bi-LSTM demonstrated a more accurate trend but still showed high noise levels. SLR performed relatively better than PCS, but SLR U-Bi-LSTM offered the most controlled pattern with minimal fluctuations. This demonstrates that the proposed U-Bi-LSTM-enhanced models are significantly more effective for extended gaps, especially in minimizing errors and aligning closely to the original data pattern.

More detailed results of comparative analyses of traditional data recovery methods such as PCS and state-of-the-art SLR, as well as U-Bi-LSTM-enhanced models, are summarized in Table S2. This table illustrates that the U-Bi-LSTM-enhanced methods, PCS U-Bi-LSTM and SLR U-Bi-LSTM, consistently demonstrated significantly lower wand length error values than the basic PCS and SLR methods across various conditions of gap length and number of gaps. To further assess the performance of the proposed enhancement in a comparative framework, the PCS U-Bi-LSTM and SLR U-Bi-LSTM methods were also evaluated against several state-of-the-art deep learning methods, specifically the bidirectional attention network (BAN) [20] and the attention-based LSTM with least squares constraint (A-LSTM+LS) [21].

**Figure 11:**
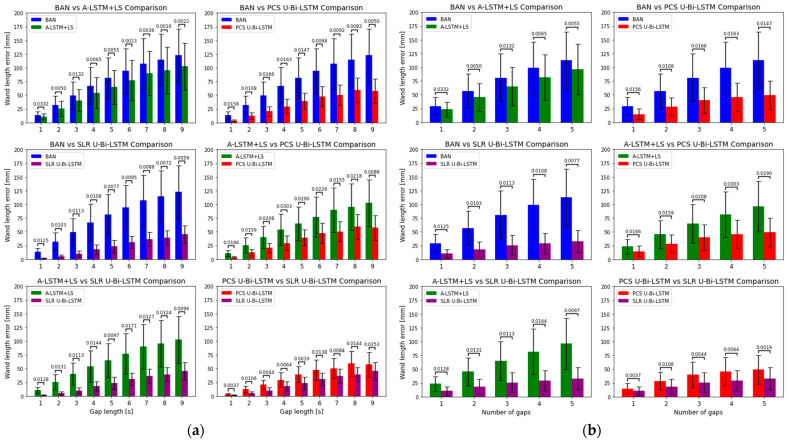
Comparative analyses of wand length error reduction by the U-Bi-LSTM enhanced and state-of-the-art MoCap data recovery deep learning methods across varied missing data gap conditions. (**a**) Wand length error comparison by gap length, and (**b**) by number of gaps.

The wand length error presented in Figure 11 reveals distinct performance differentials between the four methods—BAN, A-LSTM+LS, PCS U-Bi-LSTM, and SLR U-Bi-LSTM—across varying gap lengths and numbers of gaps. The PCS U-Bi-LSTM and SLR U-Bi-LSTM methods consistently demonstrated superior performance in reducing wand length error compared to BAN and A-LSTM+LS. This superiority is quantitatively significant, with RMSE values markedly lower for the U-Bi-LSTM enhanced models across all tested conditions. As the gap length increased from 1 s to 9 s and the number of gaps from 1 to 5, wand length error showed an increasing trend in all methods, but the proposed U-Bi-LSTM methods demonstrated a smaller increase in wand length error as the gap length and number of gaps increased compared to the state-of-the-art deep learning methods (BAN and A-LSTM+LS). Statistical analysis conducted through paired t-tests confirms that the differences in RMSEs between each pair of methods were statistically significant (*p* < 0.05). This not only suggests a genuine performance enhancement by the U-Bi-LSTM architectures but also reinforces the reliability of these findings under the varied experimental conditions. The narrower standard deviation ranges for the PCS U-Bi-LSTM and SLR U-Bi-LSTM methods further underscore their robustness and consistency in performance, a crucial attribute for practical deployment in MoCap applications where variability can greatly affect the fidelity of motion capture reconstructions. The enhanced performance of the U-Bi-LSTM models can be attributed to their architectural features. The integration of Bi-LSTM layers enables effective learning of dependencies in time-series data both forward and backward in time, a feature that is critical in accurately bridging lengthy data gaps. The U-net-inspired skip connections facilitate the preservation and integration of feature information across the network, enhancing the model’s ability to reconstruct complex motion patterns from fragmented inputs. The adaptive Huber loss function further refines the model’s error sensitivity, balancing the treatment of outliers and reducing the influence of large errors, which is particularly beneficial in scenarios with significant data gaps. In comparison, while A-LSTM+LS and BAN incorporate sophisticated learning mechanisms, their lesser performance relative to the U-Bi-LSTM models might stem from their potential limitations in effectively handling extensive and diverse data gaps. A-LSTM+LS, despite its attention mechanisms and least squares constraint, which typically aid in stabilizing the learning process, still falls short against the proposed U-Bi-LSTM-enhanced methods. This indicates that while attention mechanisms provide a focused learning capability, without the structural and loss function optimizations in the U-Bi-LSTM models, their efficacy is limited in long-term missing MoCap data recovery tasks. BAN’s performance lagging behind A-LSTM+LS could be attributed to its reliance on bidirectional attention without the regularization benefits provided by the least squares constraints, making it more susceptible to variations in data with extensive missing segments. The strong dependence of BAN and A-LSTM+LS on inter-marker relations and predefined motion models is possibly one of the main reasons for their relatively large wand length error (where there was only one reference marker) compared to the proposed U-Bi-LSTM-enhanced methods. These state-of-the-art deep learning methods often rely heavily on correlations between markers to predict missing data, which can falter in scenarios where data are available only from a single marker or where marker interactions are complex. In contrast, the PCS U-Bi-LSTM and SLR U-Bi-LSTM methods are specifically designed to effectively and independently recover long-term lost data by utilizing available multi-camera UV data in combination with XYZ marker tracking data. This approach leverages the fundamental motion capture process where UV coordinates from multiple camera views are used to triangulate the XYZ positions in 3D space. Even when complete triangulation is hindered due to missing data, the integration of available UV data from at least one camera can significantly enhance the accuracy of reconstructing lost tracking data. This method capitalizes on any available data to infer missing information more robustly than the methods relying solely on inter-marker relational models or available XYZ data, providing a resilient framework against scenarios with incomplete data. This is particularly advantageous in complex MoCap environments where precise tracking is critical and long-term data loss may frequently occur.

Therefore, the proposed methods not only show lower mean reconstruction errors but also show reduced variability, as evidenced by the narrower confidence intervals. The results of the statistical analyses reinforce these observations, confirming that the performance enhancement is not sporadic but consistently substantial across all experimental motions and recovery scenarios. In essence, the integration of U-net-inspired skip connections, adaptive Huber loss, and bidirectional learning into the U-Bi-LSTM framework results in a methodologically solid and empirically robust approach. This novel framework has proven its efficacy through rigorous testing, and its effects are validated by substantial reductions in error metrics, thus affirming its capacity for precise and dependable MoCap data recovery across the board.

While the enhanced methods demonstrated remarkable performance, certain limitations were also identified:

- Camera layout and positioning sensitivity. The efficacy of the proposed methods relies heavily on precise multicamera UV data as well as triangulated X, Y, Z data, making it highly sensitive to nuances in camera positioning and layout. The high accuracy in recovering lost tracking data demonstrated by the method proposed in this study presupposes an environment where cameras are correctly positioned, thus enabling rigorous multi-camera calibration and reliable object tracking. Any deviation from the recommended setup [4,5] would necessitate a recalibration of the system and potentially retraining of the algorithm, which may pose challenges when applied in practice in varied environments that differ from the initial configuration. Moreover, real-world applications might encounter physical or logistical constraints that preclude ideal camera placements. Consequently, future research could focus on devising more flexible models that can autonomously adapt to camera layout changes. This could be achieved by using advanced machine learning techniques that learn to discern and adjust to the most effective configurations based on feedback from the environment [47].
- Impact of environmental changes on model performance. Environmental factors such as lighting variations and obstructions pose significant challenges to the consistency of MoCap data quality. The proposed model was developed in controlled laboratory conditions with carefully curated datasets, enabling us to isolate and focus on recovering lost tracking data. This does not yet account for more nuanced disturbances like noise or marker mismatches. Hence, the model’s resilience to environmental perturbations must be comprehensively evaluated before it can be successfully implemented in real-world applications. Future research directions could include the integration of pre-processing layers capable of identifying and compensating for such environmental influences, thus enhancing the model’s overall robustness [48,49].
- Dependence on extensive training data. This model’s reliance on a large volume of high-quality training data remains a notable limitation. While data augmentation strategies are employed in an attempt to mitigate this issue, the collection of diverse and comprehensive datasets is crucial for the continued success and improvement of these models. In future research, exploring generative models for synthesizing MoCap data under varied conditions [50,51] could significantly alleviate the constraints imposed by the need for large training datasets.

## 4. Conclusions

In conclusion, the present work has empirically validated that introducing U-Bi-LSTM to traditional PCS and state-of-the-art SLR methods leads to significant advancements for independent long-term missing data recovery in motion capture. Compared to previous approaches, our model tackles inherent challenges by adapting to varying conditions through its bidirectional nature, U-net-inspired skip connections, and adaptive Huber loss. These features, among others, allow the model to handle long-term gaps and maintain data integrity. Quantitative analyses demonstrated marked improvements in missing data recovery error values across various types and intensities of motion, gap lengths, and numbers of gaps, with statistical analyses confirming the statistical significance of these results. These findings not only encourage the use of PCS U-Bi-LSTM and SLR U-Bi-LSTM as robust solutions for motion capture data integrity, but they also pave the way for further research to continue refining and adapting these deep-learning-based techniques for broader applications in motion analysis.

## Figures and Tables

**Table 1:** Optimized hyperparameters of the PCS U-Bi-LSTM and SLR U-Bi-LSTM.

	Training Option	Range of Parameters	PCS U-Bi-LSTM Selected Parameters	SLR U-Bi-LSTM Selected Parameters
1	Input normalization	[zero-center, zscore, rescale-symmetric, rescale-zero-one]	zscore	rescale-zero-one
2	Input length	[10, 50, 100, 1000]	10	10
3	Adaptive Huber alpha	[0.0001, 0.001, 0.01, 1, 1.345]	0.01	0.01
4	Maximum epoch	[5, 10, 20, 30, 40, 50]	5	5
5	Mini batch size	[8, 16, 32, 64]	32	64
6	Optimizer	[sgdm, rmsprop, adam]	adam	adam
7	Initial learning rate	[1 × 10^−6^, 1 × 10^−5^, 1 × 10^−4^, 1 × 10^−3^, 1 × 10^−2^]	0.001	0.001
8	Learning rate drop period	-	10	10
9	Learning rate drop factor	-	0.2	0.2

## Data Availability

The data that support the findings of this study are available on request from the corresponding author. The data are not publicly available due to privacy or ethical restrictions.

## Supplementary Materials

The following supporting information can be downloaded at https://www.mdpi.com/article/10.3390/bioengineering11060560/s1. Table S1: Results of an ablation study of the proposed U-Bi-LSTM architecture on motion capture missing data reconstruction error across various gap lengths and numbers. The results of the architecture with the smallest reconstruction error are highlighted in bold; Table S2: Comparative analysis of wand length error (RMSE+SD) [mm] using the traditional PCS, state-of-the-art SLR, and the proposed PCS U-Bi-LSTM and SLR U-Bi-LSTM with different gaps lengths and numbers. The results of the method with the smallest wand length error are highlighted in bold.
