# Supplementary material for: Deep-Learning-Based Recovery of Missing Optical Marker Trajectories in 3D Motion Capture Systems
Source: Bioengineering (Basel). 2024 Jun 1;11(6):560. doi: 10.3390/bioengineering11060560 (PMC11200691; doi:10.3390/bioengineering11060560)
Supplement: Supplementary file 1 [file bioengineering-11-00560-s001.zip › bioengineering-3003858-supplementary.pdf]

Deep-Learning-Based Recovery of Missing Optical Marker Trajectories in 3D Motion Capture Systems

Oleksandr Yuhai [1], Ahnryul Choi [2], Yubin Cho [1], Hyunggun Kim [1] and Joung Hwan Mun [1]

[1]Department of Bio-Mechatronic Engineering, College of Biotechnology and Bioengineering, Sungkyunkwan University, Republic of Korea
[2]Department of Biomedical Engineering, College of Medical Convergence, Catholic Kwandong University, Republic of Korea

Co-corresponding author:
Hyunggun Kim, Ph.D.
Department of Bio-Mechatronic Engineering
College of Biotechnology and Bioengineering
Sungkyunkwan University
2066 Seoburo, Jangan, Suwon, Gyeonggi, 16419, Republic of Korea
Email: hkim.bme@skku.edu

Corresponding author:
Joung Hwan Mun, Ph.D.
Department of Bio-Mechatronic Engineering
College of Biotechnology and Bioengineering
Sungkyunkwan University
2066 Seoburo, Jangan, Suwon, Gyeonggi, 16419, Republic of Korea
Email: jmun@skku.edu

Table S1. Results of an ablation study of the proposed U-Bi-LSTM architecture on motion capture missing data reconstruction error across various gap lengths and numbers. The results of the architecture with the smallest reconstruction error are highlighted in bold.

		Gap length								
Architecture	Number of gaps	1s	2s	3s	4s	5s	6s	7s	8s	9s
Bi-LSTM + RMSE regression loss	1 gap	62.41 ± 3.98	89.95 ± 7.39	106.93 ± 5.31	123.24 ± 8.67	144.08 ± 7.93	157.08 ± 11.68	159.69 ± 5.24	177.49 ± 3.91	186.53 ± 7.10
	2 gaps	87.56 ± 4.90	125.82 ± 7.26	151.23 ± 5.59	177.41 ± 4.18	199.42 ± 4.88	218.34 ± 8.21	237.65 ± 4.06	249.17 ± 5.81	266.53 ± 5.52
	3 gaps	109.12 ± 4.21	154.56 ± 5.37	188.98 ± 4.59	214.97 ± 9.64	227.86 ± 11.73	263.95 ± 9.58	275.42 ± 7.35	292.08 ± 13.56	307.878± 8.28
	4 gaps	126.10 ± 4.92	176.80 ± 7.61	218.37 ± 4.96	249.91 ± 7.48	273.09 ± 8.05	289.89 ± 6.93	313.37 ± 15.39	328.17 ± 10.80	330.76 ± 11.48
	5 gaps	139.32 ± 5.03	199.86 ± 5.42	239.39 ± 4.90	278.45 ± 5.34	303.73 ± 7.42	320.87 ± 9.20	341.25 ± 7.91	350.92 ± 12.54	369.09 ± 14.18
Bi-LSTM + Adaptive Huber loss	1 gap	33.02 ± 3.949	49.84 ± 7.26	60.035 ± 5.22	70.84 ± 8.67	72.57 ± 7.89	82.95 ± 11.62	94.27 ± 4.88	101.66 ± 3.82	106.89 ± 5.82
	2 gaps	47.65 ± 4.71	69.67 ± 7.22	86.57 ± 4.93	106.83 ± 3.86	110.08 ± 4.58	123.19 ± 7.81	126.82 ± 3.17	138.20 ± 5.64	143.45 ± 5.46
	3 gaps	63.43 ± 3.79	85.48 ± 4.82	100.46 ± 3.98	120.92 ± 8.78	130.36 ± 8.93	148.12 ± 5.15	160.34 ± 6.94	163.16 ± 8.03	174.64 ± 5.34
	4 gaps	69.02 ± 4.34	95.24 ± 7.42	119.00 ± 3.99	130.97 ± 6.72	146.73 ± 7.72	158.97 ± 5.87	171.68 ± 6.08	180.97 ± 7.84	186.85 ± 7.34
	5 gaps	75.94 ± 4.48	110.72 ± 5.28	129.85 ± 4.53	149.53 ± 5.29	161.86 ± 6.83	176.97 ± 9.04	189.53 ± 7.66	197.24 ± 7.33	202.45 ± 7.41
Bi-LSTM + U-net component + RMSE regression loss	1 gap	32.60 ± 3.98	49.34 ± 7.31	59.61 ± 5.36	70.30 ± 8.71	71.58 ± 8.19	79.44 ± 6.95	93.12 ± 4.74	100.82 ± 3.70	105.88 ± 5.24
	2 gaps	47.03 ± 4.70	68.90 ± 7.13	85.61 ± 4.80	106.10 ± 3.85	108.28 ± 3.81	122.25 ± 7.84	126.01 ± 2.34	137.39 ± 5.29	142.75 ± 5.40
	3 gaps	62.91 ± 3.68	85.01 ± 3.88	99.75 ± 3.91	119.94 ± 8.76	129.68 ± 8.25	147.13 ± 4.15	159.67 ± 6.83	162.44 ± 7.99	173.85 ± 5.29
	4 gaps	68.54 ± 4.19	94.47 ± 7.22	118.26 ± 3.73	130.25 ± 6.66	145.73 ± 7.61	158.16 ± 5.81	170.95 ± 6.09	179.34 ± 6.86	186.42 ± 7.14
	5 gaps	75.349 ± 4.05	109.98 ± 5.12	128.93 ± 5.24	148.86 ± 5.15	161.08 ± 6.49	176.28 ± 8.89	188.78 ± 7.47	196.24 ± 7.31	200.57 ± 7.14
**Bi-LSTM + U-net component + Adaptive Huber loss**	1 gap	**21.43 ± 3.88**	**36.06 ± 7.03**	**42.93 ± 4.63**	**53.26 ± 5.23**	**53.54 ± 7.48**	**59.15 ± 7.65**	**68.16 ± 4.65**	**70.26 ± 3.90**	**78.34 ± 5.16**
	2 gaps	**35.15 ± 4.69**	**49.56 ± 4.09**	**60.32 ± 4.65**	**78.19 ± 3.46**	**78.51 ± 3.68**	**90.49 ± 7.37**	**89.01 ± 2.10**	**100.28 ± 5.14**	**102.40 ± 5.36**
	3 gaps	**44.68 ± 3.28**	**59.25 ± 3.41**	**74.34 ± 3.58**	**89.33 ± 5.98**	**93.66 ± 7.44**	**108.83 ± 4.03**	**117.55 ± 8.75**	**119.03 ± 7.86**	**129.49 ± 5.16**
	4 gaps	**51.07 ± 3.34**	**67.61 ± 6.65**	**86.57 ± 3.71**	**94.45 ± 4.44**	**109.01 ± 6.77**	**112.63 ± 5.63**	**124.44 ± 5.76**	**126.49 ± 5.85**	**132.51 ± 6.68**
	5 gaps	**54.74 ± 3.87**	**80.30 ± 4.24**	**95.74 ± 5.12**	**111.31 ± 5.02**	**115.03 ± 6.03**	**127.63 ± 8.25**	**136.99 ± 6.97**	**143.64 ± 6.15**	**149.63 ± 6.09**

Table S2. Comparative analysis of wand length error (RMSE+SD) [mm] using the traditional PCS, state-of-the-art SLR, and the proposed PCS U-Bi-LSTM and SLR U-Bi-LSTM with different gaps lengths and numbers. The results of the method with the smallest wand length error are highlighted in bold.

		Gap length								
Architecture	Number of gaps	1s	2s	3s	4s	5s	6s	7s	8s	9s
PCS	1 gap	2.37 ± 0.62	10.22 ± 3.55	27.72 ± 7.14	53.16 ± 21.2	91.04 ± 33.34	98.73 ± 18.1	151.18 ± 18.3	197.74 ± 21.77	200.68 ± 46.23
	2 gaps	5.27 ± 0.76	23.27 ± 2.98	59.76 ± 17.26	86.93 ± 22.93	185.01 ± 21.27	243.47 ± 32.31	285.5 ± 33.74	390.24 ± 53.76	438.33 ± 38.81
	3 gaps	8.68 ± 1.08	31.75 ± 8.22	81.46 ± 6.08	164.57 ± 30.88	280.85 ± 44.88	369.15 ± 61.85	426.95 ± 59.43	525.83 ± 13.04	560.86 ± 62.07
	4 gaps	10.92 ± 2.01	49.17 ± 6.11	92.89 ± 16.26	209.29 ± 15.64	374.66 ± 47.6	423.72 ± 33.12	537.53 ± 46.5	788.77 ± 63.05	919.77 ± 102.32
	5 gaps	12.46 ± 1.53	60.57 ± 9.59	134.57 ± 17.06	279.46 ± 40.8	415.24 ± 26.29	552.47 ± 97.08	694.99 ± 59.26	768.37 ± 41.48	996.63 ± 159.93
PCS U-Bi-LSTM	1 gap	1.29 ± 0.52	4.6 ± 2.45	7.54 ± 2.24	9.91 ± 2.53	16.51 ± 6.19	15.81 ± 6.36	25.4 ± 6.51	25.19 ± 6.69	29.34 ± 7.46
	2 gaps	2.6 ± 0.62	8.46 ± 2.01	16.65 ± 2.32	24.87 ± 5.82	28.39 ± 4.54	35.58 ± 7.94	42.08 ± 6.23	47.23 ± 12.87	62.4 ± 12.42
	3 gaps	4.42 ± 0.35	12.04 ± 2.72	20.46 ± 4.26	32.39 ± 5.65	38.12 ± 4.73	56.17 ± 5.29	59.24 ± 7.28	62.89 ± 8.09	63.89 ± 8.54
	4 gaps	5.09 ± 1.09	14.83 ± 2.03	24.65 ± 3.74	42.62 ± 3.85	49.69 ± 9.22	47.48 ± 5.49	59.52 ± 8.24	65.37 ± 9.64	70.7 ± 11.68
	5 gaps	6.74 ± 0.96	21.99 ± 1.91	32.41 ± 5.91	53.29 ± 6.65	54.85 ± 8.36	56.74 ± 9.43	70.14 ± 6.66	71.06 ± 14.68	74.76 ± 20.82
SLR	1 gap	1.18 ± 0.21	2.94 ± 0.41	4.97 ± 0.62	7.56 ± 1.41	15.46 ± 2.47	20.26 ± 5.8	29.3 ± 6.19	38.59 ± 5.88	39.38 ± 7.99
	2 gaps	2.18 ± 0.26	6.13 ± 0.61	11.98 ± 2.21	20.51 ± 4.68	33.98 ± 3.64	43.27 ± 7.01	42.35 ± 6.09	51.12 ± 11.38	64.01 ± 6.81
	3 gaps	3.24 ± 0.33	8.23 ± 0.62	17.7 ± 2.24	26.6 ± 3.18	41.95 ± 3.94	66.78 ± 5.13	64.36 ± 6.36	61.95 ± 8.02	78.53 ± 10.23
	4 gaps	3.82 ± 0.34	11.17 ± 0.69	19.44 ± 3.33	38.1 ± 3.42	55.32 ± 9.14	65.67 ± 5.12	58.74 ± 8.18	65.72 ± 9.11	75.03 ± 10.78
	5 gaps	4.57 ± 0.82	13.9 ± 1.66	27.68 ± 3.75	42.74 ± 3.99	57.83 ± 7.75	57.17 ± 6.39	84.83 ± 5.92	80.38 ± 12.89	87.43 ± 19.28
**SLR U-Bi-LSTM**	1 gap	**0.79 ± 0.21**	**1.99 ± 0.37**	**3.39 ± 0.46**	**4.7 ± 0.91**	**8.56 ± 2.19**	**11.84 ± 2.02**	**16.23 ± 5.47**	**21.47 ± 2.42**	**21.46 ± 6.37**
	2 gaps	**1.42 ± 0.29**	**3.43 ± 0.47**	**6.91 ± 0.66**	**12.48 ± 2.28**	**25.07 ± 3.39**	**27.91 ± 4.74**	**31.01 ± 5.63**	**32.65 ± 10.85**	**37.31 ± 6.36**
	3 gaps	**2.13 ± 0.31**	**4.89 ± 0.32**	**11.22 ± 1.63**	**16.01 ± 2.86**	**23.79 ± 3.58**	**42.64 ± 4.45**	**39.77 ± 3.71**	**40.97 ± 7.07**	**39.08 ± 9.89**
	4 gaps	**2.53 ± 0.36**	**6.48 ± 0.92**	**11.9 ± 1.81**	**24.5 ± 3.22**	**33.82 ± 9.05**	**41.23 ± 5.06**	**40.43 ± 6.39**	**43.51 ± 5.03**	**45.12 ± 10.11**
	5 gaps	**3.26 ± 0.11**	**8.61 ± 1.48**	**15.11 ± 2.04**	**28.65 ± 3.51**	**34.74 ± 4.71**	**36.23 ± 5.47**	**52.33 ± 3.9**	**44.1 ± 3.07**	**53.99 ± 11.02**
